# Population Nutritional Status in Addis Health and Demographic Surveillance System (ADDIS-HDSS), Addis Ababa, Ethiopia

**DOI:** 10.4314/ejhs.v34i2.7S

**Published:** 2024-12

**Authors:** Tigest Shifraw, Semira Abdelmenan, Hanna Yemane Berhane, Hanna Gulema, Seada Beyan, Firehiwot Workneh, Kalkidan Yibeltal, Sitota Tsegaye, Gadise Bekele, Nebiyou Fasil, Dongqing Wang, Uttara Partap, Christopher Robert Sudfeld, Wafaie Fawzi, Meaza Demissie, Alemayehu Worku, Yemane Berhane

**Affiliations:** 1 Department of Reproductive Health and Population, Addis Continental Institute of Public Health; 2 Department of Epidemiology and Biostatistics, Addis Continental Institute of Public Health; 3 Department of Nutrition and Behavioral Science, Addis Continental Institute of Public Health; 4 Department of Global Health and Health Policy, Addis Continental Institute of Public Health; 5 Department of Global and Community Health, College of Public Health, George Mason University, Fairfax, Virginia, United States of America; 6 Department of Global Health and Population, Harvard T.H. Chan School of Public Health, Harvard University, Boston, Massachusetts, United States of America

**Keywords:** Underweight, overweight/obesity, nutritional status, children, adolescents, adults, Ethiopia

## Abstract

**Background:**

Low—and middle-income countries face a double burden of malnutrition. However, comprehensive, population-based nutritional assessments are rare, particularly across all age groups. This study aimed to assess the prevalence of malnutrition across different age groups in Addis Ababa Health and Demographic Surveillance Site (Addis-HDSS), Addis Ababa, Ethiopia.

**Methods:**

A community-based cross-sectional survey was conducted from December 2022 to January 2023 in Addis Ababa, involving residents of the Addis-HDSS sites. Mid-upper arm circumference (MUAC) was used to assess nutritional status for individuals aged 6 months to 64 years. Descriptive statistics were analyzed using STATA version 14, employing previously published age-specific cutoff points to define underweight, overweight, and obesity.

**Results:**

A total of 37,364 individuals aged 6 months to 64 years participated. Among children aged 6-59 months, 4.2% had moderate acute malnutrition (95% CI: 3.7-4.9), and 3.0% had severe acute malnutrition (95% CI: 2.5-3.6). Overweight prevalence was 22.3% (95% CI: 20.3-24.3) in children aged 5-9 years, 25.9% (95% CI: 23.4-28.5) in adolescents aged 10-14 years, and 12.7% (95% CI: 11.5-14.0) in late adolescents aged 15-19 years. Among adults aged 20-64 years, 6.3% were underweight (95% CI: 6.0-6.6), 19.3% overweight (95% CI: 18.8-19.7%), and 21.5% obese (95% CI: 21.0-22.0).

**Conclusions:**

This study highlights a double burden of malnutrition in Addis Ababa, with overweight and obesity more prevalent than underweight, especially in adolescents and adults. It underscores the need for interventions targeting both undernutrition and overnutrition, emphasizing better diets and physical activity to curb nutrition-related diseases.

## Introduction

Nutrition plays a critical role in maintaining health, preventing diseases, and ensuring optimal growth and development (1,2). In low- and middle-income countries (LMICs), malnutrition manifests in various forms, including undernutrition, overweight, obesity, and micronutrient deficiencies. The co-occurrence of undernutrition and overnutrition within the same population is referred to as the “double burden of malnutrition” (DBM), which is becoming increasingly common in LMICs (3). This shift reflects stagnation or slow progress in combating undernutrition alongside rapid increases in overweight and obesity, particularly in urban settings (4).

Malnutrition has detrimental effects across the life course. Undernutrition is associated with stunted growth, weakened immune function, and higher susceptibility to infectious diseases, while overweight and obesity contribute to the development of non-communicable diseases (NCDs) such as diabetes, cardiovascular diseases, and certain cancers (5–7). The double burden of malnutrition is especially concerning, as it contributes to both immediate and long-term health challenges.

This study sought to assess the prevalence of malnutrition across various age groups in Addis Ababa, Ethiopia, to provide data that could inform public health policy, nutritional interventions, and future research on malnutrition trends in the country.

## Methods

**Study design and participants**: A community-based cross-sectional survey was conducted from December 2022 to January 2023 in Addis Ababa, at the Addis-HDSS site, which monitors residents' health and demographic status in the Yeka sub-city. A total of 37,364 individuals aged 6 months to 64 years were included in the study.

**Nutritional assessment**: Mid-upper arm circumference (MUAC) was measured to assess nutritional status. MUAC is a simple and cost-effective method to determine undernutrition, overweight, and obesity (8,9). Age-specific MUAC cutoff points, based on established international and Ethiopian guidelines, were used to classify individuals as underweight, overweight, or obese (8,10–14) (Table 1).

**Table 1:** MUAC cutoffs from 6 months to 64 years

Author, Year	Age category	SAM	MAM	Normal	Overweight	
WHO classification of acute malnutrition	6-59 months	<11.5 cm	≥11.5 cm to <12.5 cm	≥12.5 cm	N/A	
Craig E, 2014 Cashen K & Oat L, 2018	5-9 years	<13.5 cm	≥13.5 cm to <14.5 cm	≥14.5 cm	Boys: >18.4	Girls: >18.3
	10-14 years	<16.0 cm	≥16.0 cm to <18.0 cm	≥18.0 cm	Male: >23.2	Female: >22 cm
Sisay BG, 2020 & 2023	15-19 years	<18.0 cm	≥18.0 cm to ≤22.5 cm	>22.5 cm to <27.7 cm (Male) >22.5 cm to <27.9 cm (Female)	Male: ≥27.7 cm	Female: ≥27.9 cm
	**Adults**	**Underweight**	**Normal**	**Overweight**	**Obesity**	
ThorupL, 2023 & Shifraw T, 2021	20-64 years	<24.5 cm	≥24.5 cm to ≤28.0 cm	>28.0 cm to ≤30.0 cm	>30 cm	

SAM: Severe Acute Malnutrition, MAM: Moderate Acute Malnutrition, N/A: Not available (MUAC only cutoff)

**Data collection and analysis**: Trained enumerators collected data using electronic devices (ODK). The data were analyzed using STATA version 14 to calculate descriptive statistics, including the prevalence of undernutrition and overnutrition across different age groups.

## Results

**Demographics**: Of the 37,364 participants, 69.9% were female, and the majority were adults aged 25-44. Among children under five, 51.4% were male, and the majority were between the ages of 24 months and 5 years (Table 2).

**Table 2:** Age and Sex characteristics of study participants in ADDIS-HDSS sites, Addis Ababa, Ethiopia (n=37,364)

Age	Female n (%)	Male n (%)	Total n (%)
6-23 months	702 (47.3)	782 (52.7)	1484 (4.0)
24-59 months	1290 (49.4)	1321 (50.6)	2611 (7.0)
5-9 years	795 (49.1)	827 (50.9)	1622 (4.3)
10-14 years	704 (61.1)	450 (38.9)	1154 (3.1)
15-19 Years	2111 (77.9)	599 (22.1)	2710 (7.3)
20-24 years	3356 (77.3)	988 (22.7)	4344 (11.6)
25-44 years	12835 (74.1)	4506(25.9)	17341 (46.4)
45-64 Years	4315 (70.8)	1783 (29.2)	6098 (16.3)
Total	26108(69.9)	11256 (30.1)	37364 (100.0)

**Malnutrition prevalence among children (6-59 months)**: The prevalence of moderate acute malnutrition was 4.2%, and severe acute malnutrition was 3.0%. Acute malnutrition was more common in children aged 6-23 months (10.9%) compared to those aged 24-59 months (5.2%) (Table 3).

**Table 3:** Nutritional status of children aged 6-59 months in ADDIS-HDSS, Addis Ababa, Ethiopia (n=4095)

Nutritional status	6 to 23 months n (%)	24-59 months n (%)	Under five-year (6 - 59 months) n (%)
Normal	1323 (89.2)	2476 (94.8)	3799 (92.8)
Female	625 (42.1)	1219 (46.7)	1844 (45.0)
Male	698 (47.0)	1257 (48.1)	1955 (47.7)
Moderate undernutrition	89 (6.0)	84 (3.2)	173 (4.2)
Female	40 (2.7)	49 (1.9)	89 (2.2)
Male	49 (3.3)	35 (1.3)	84 (2.1)
Severe undernutrition	72 (4.9)	51 (2.0)	123 (3.0)
Female	37 (2.5)	22 (0.8)	59 (1.4)
Male	35 (2.4)	29 (1.1)	64 (1.6)

**Malnutrition prevalence among children and adolescents (5-19 years)**: Among children aged 5-9 years, 22.3% were overweight. Among adolescents aged 10-14 years, the prevalence of overweight was higher at 25.9%. For late adolescents (15-19 years), 12.7% were overweight, with females exhibiting a higher prevalence than males (Table 4).

**Table 4:** Nutritional status of adolescents aged 5– 19 years in ADDIS-HDSS Addis Ababa, Ethiopia. (n=5486)

Nutritional status	5-9 Years n (%)	10-14 years n (%)	15-19 years n (%)
Normal	1114 (68.7)	531 (46.0)	1782 (65.8)
Female	531 (32.7)	339 (29.4)	1435 (53.0)
Male	583 (35.9)	192 (16.6)	347 (12.8)
Moderate undernutrition	85 (5.2)	273 (23.7)	566 (20.9)
Female	38 (2.3)	133 (11.5)	416 (15.4)
Male	47 (2.9)	140 (12.1)	150 (5.5)
Severe undernutrition	62 (3.8)	51 (4.4)	18 (0.7)
Female	32 (2.0)	22 (1.9)	14 (0.5)
Male	30 (1.8)	29 (2.5)	4 (0.2)
Overweight	361 (22.3)	299 (25.9)	344 (12.7)
Female	194 (12.0)	210 (18.2)	246 (9.1)
Male	167 (10.3)	89 (7.7)	98 (3.6)

**Figure 1:**
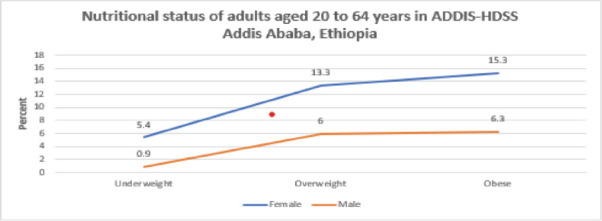
Nutritional status of adults aged 20-64 years in ADDIS-HDSS Addis Ababa, Ethiopia

**Malnutrition prevalence among adults (20-64 years)**: The prevalence of underweight among adults was 6.3%. However, the prevalence of overweight and obesity was notably higher: 19.3% and 21.5%, respectively (Table 5). The gender disparity was marked, with females having higher rates of obesity (15.3%) compared to males (6.3%) (Figure 1).

**Table 5:** Nutritional status of adults categorized by age in ADDIS-HDSS Addis Ababa, Ethiopia. (n=27,783)

Nutritional status	20-24 Years n (%)	25-44 years n (%)	45-64 years n (%)
Normal	2997 (69.0)	9081 (52.4)	2615 (42.9)
Female	2319 (53.4)	6878 (39.7)	1863 (30.6)
Male	678 (15.6)	2203 (12.7)	752 (12.3)
Underweight	569 (13.1)	941 (5.4)	243 (4.0)
Female	513 (11.8)	809 (4.7)	189 (3.1)
Male	56 (1.3)	132 (0.8)	54 (0.9)
Overweight	522 (12.0)	3496 (20.2)	1340 (22.0)
Female	355 (8.2)	2422 (14.0)	920 (15.1)
Male	167 (3.8)	1074 (6.2)	420 (6.9)
Obesity	256 (5.9)	3823 (22.0)	1900 (31.1)
Female	169 (3.9)	2726 (15.7)	1343 (22.0)
Male	87 (2.0)	1097 (6.3)	557 (9.1)

## Discussion

This study highlights the emerging double burden of malnutrition in Addis Ababa, with a strikingly higher prevalence of overweight and obesity than underweight across various age groups. The prevalence of acute malnutrition among children was relatively low but still concerning, especially in younger children. The high rates of overweight in both children and adolescents suggest a nutritional transition, likely driven by urbanization, dietary changes, and reduced physical activity.

The severe acute malnutrition (SAM) rate for Addis Ababa, according to the Ethiopian Demographic Health Survey (EDHS) 2019, was 3%, which is comparable to this study (15), and it was lower than that reported in the Seqota district Wag Hemera zone (8.0%) (16) and the South Gondar Zone (11.2%) in the Amhara region of Ethiopia (17). The lower prevalence compared to other studies may be because this study was conducted in an urban population with universal access to primary education, improved household socioeconomic status, adequate maternal healthcare utilization, and awareness about nutrition (18), whereas the above two studies were conducted in predominantly food-insecure rural areas. The prevalence of SAM was higher among 6- to 23-month-old children than older children (24- 59 months), which could be related to the damage done in the first 1000 days of a child's life (15).

The highest prevalence of overweight was observed in children and adolescents aged 5 to 19; this is consistent with the figure reported by a meta-analysis, which also highlighted that the highest prevalence was in Addis Ababa compared to other regions of the country (19,20). This could be due to better access to high-calorie diets in the capital than in other areas of the country. In support of this, an in-school study reported that sweets and sugar-sweetened beverages, as well as deep-fried food, were commonly consumed by adolescents (21,22). Further, the past two decades have also seen an increase in mobile and gaming devices, leading to increased mindless eating while watching screens, decrement in physical activities, increased sedentary behaviors, and reduced sleep time (23). Overweight/obesity is now emerging as a public health concern in low- and middle-income countries (LMICs), contrary to previous concerns that focused primarily on undernutrition. This study indicates an ongoing nutrition transition, at least in urban areas of the country (24).

The risk of developing overweight among female adults was higher than among males in this study. The result was similar to studies conducted in African countries (25). The biological difference between males and females in energy requirements may explain our finding (26). Furthermore, males are more physically active as they are more accessible to experience outdoor activities than females in LMICs (23,27), which ultimately causes overweight and obesity in girls (24).

The prevalence of double burden of malnutrition with a higher prevalence of underweight and overweight among females than males. Among adults, the highest prevalence of overweight/obesity was observed in the age group 25-44 and 45-64 years. This is aligned with global statistics, where the global burden of overweight and obesity is higher among women than men (28,29). The prevalence of overweight/obesity exceeded that of underweight. The high prevalence of overweight and obesity is a growing concern in LMICs, as it has been linked to various non-communicable diseases such as diabetes, cardiovascular disorders, and certain types of cancer (30–32). Interventions that promote healthy eating habits and physical activity are essential to addressing the issue of overweight within the community.

The study's primary limitation is that it did not assess potential factors influencing nutritional status, such as dietary habits, physical activity, or socioeconomic factors. Additionally, while MUAC is a useful tool for screening, it may not be as effective for adolescents due to changes in muscle and fat distribution.

In conclusion, this study provides evidence of the double burden of malnutrition in Addis Ababa, with a higher prevalence of overweight and obesity compared to underweight, particularly among children, adolescents, and adults. Addressing both undernutrition and overnutrition through comprehensive public health interventions is crucial to improving the overall nutritional status and preventing the rise of non-communicable diseases in Ethiopia.
